# A Pilot Randomized Controlled Trial of a Telenutrition Weight Loss Intervention in Middle-Aged and Older Men with Multiple Risk Factors for Cardiovascular Disease

**DOI:** 10.3390/nu11020229

**Published:** 2019-01-22

**Authors:** Melissa Ventura Marra, Christa L. Lilly, Kelly R. Nelson, Dominick R. Woofter, James Malone

**Affiliations:** 1 Division of Animal and Nutritional Sciences, West Virginia University, Morgantown, WV 26506, USA; 2 Department of Biostatistics, School of Public Health, West Virginia University, Morgantown, WV 26506, USA; cllilly@hsc.wvu.edu; 3 United Hospital Center, West Virginia University Medicine, Bridgeport, WV 26330, USA; krn@nelsonwv.com (K.R.N.); jemdd@aol.com (J.M.); 4 Mountain State Medical Specialties, Clarksburg, WV 26301, USA; drwoofter@hotmail.com

**Keywords:** obesity, telenutrition, telehealth, weight loss, primary care, men

## Abstract

Overweight and obesity threaten the health, functionality and quality of life of 77.2% men in West Virginia. The purpose of this study was to evaluate the feasibility and effectiveness of a 12-week primary care referred telenutrition weight loss intervention. Fifty-nine 40–70-year-old men with obesity were randomized to either the intervention group (*n* = 29) or an enhanced usual care (EUC) (*n* = 30) group. Participants from both groups were prescribed a moderate energy restricted diet (500–750 kcal/day below energy requirements) and provided diet-related educational materials; but, only those in the intervention group received weekly support from a registered dietitian nutritionist via telephone and videoconferencing. Both groups significantly reduced body weight, waist circumference, percent body fat and caloric intake and improved diet quality from baseline (*p* < 0.0001). Groups did not differ after controlling for time (all *p* > 0.30) and none of the group by time interactions were statistically significant. At week 12, a greater proportion of participants from the intervention group than the EUC group lost at least 5% of their baseline weight, (70.4% vs. 41.4%, *p* = 0.035). Retention rates and participant-reported adherence and satisfaction rates were ≥80% in the telenutrition group, thereby meeting the a priori criterion for feasibility of a larger trial. Primary care referred telenutrition interventions have the potential to improve access to dietary counseling for obesity treatment in health disparate populations. A larger longer-term trial is warranted.

## 1. Introduction

Overweight and obesity threaten the health, functionality and quality of life of 70.7% of men and 58.8% of women in the United States (U.S.) [1]. Health care costs associated with obesity and related chronic conditions such as cardiovascular disease (CVD) and diabetes burden families and health care systems [2]. Thus, secondary prevention of chronic disease and disability remains an urgent public health matter in the U.S.—especially in West Virginia (WV) where rates of potentially preventable diet-related conditions are the highest in the nation. West Virginians rank first in the U.S. in rates of obesity (37.7%), hypertension (42.7%) and diabetes (15.0%) [3]. In WV, middle-aged adults (45–64-year-olds) including most of the Baby Boomer generation, have higher obesity rates than any other age group within the state (45.0%) [3], and they rank first when compared to middle-aged adults across all states [4]. Three of every four men in WV are overweight or obese. Despite the high prevalence of overweight and obesity among men, weight loss interventions have predominantly been evaluated in women. A systematic review reported that only around 27% of participants in weight loss studies were men and only around 4% of weight-loss studies include only men [5]. Taken together, middle-aged men in WV are disproportionately affected by obesity and represent an understudied subpopulation at-risk for costly weight-related comorbidities. Clinical care guidelines [6] and U.S. Preventative Services Task Force (USPSTF) recommendations [7] identify diet and behavioral counseling as essential components of obesity treatment in primary care. Weight loss as low as 3–5% of baseline weight can result in clinically significant reductions in CVD risk. However, delivery in primary care is limited by competing demands on the primary care practitioner (PCPs) time, lack of behavioral counseling skills and lack of resources to deliver on-site intensive comprehensive programs [8,9]. PCPs have identified registered dietitian nutritionist (RDNs) as the most qualified providers of nutrition care for patients with obesity [10] but referrals are impeded by lack of direct payment for intensive counseling and poor provider access in rural areas like WV [11,12]. Increased access to nutrition care including diet-related counseling is needed to translate treatment guidelines and policy recommendations into practice. One potential solution to overcoming access barriers in rural areas is telenutrition, the provision of evidence-based Medical Nutrition Therapy (MNT) by an RDN using interactive electronic information and telecommunications technology such as videoconferencing with patients at a remote location [13]. An Academy of Nutrition and Dietetics’ expert workgroup concluded that delivering weight loss treatment using combinations of in-person and telenutrition care is as effective as in-person care but use of solely non-face-to-face telenutrition interventions for weight loss is not adequately researched [14].

We conducted a randomized controlled pilot study to determine the effects of a telenutrition weight loss program in a middle-aged and older male Appalachian population in WV. The purpose of the study was (1) to evaluate feasibility and acceptability in terms of recruitment, retention, adherence and satisfaction and (2) to assess the short-term effectiveness of the male-only telenutrition program compared to enhanced usual care regarding changes in primary (weight) and secondary (percent body fat, waist circumference, energy intake and diet quality) outcome measures. We would deem the program feasible for a larger trial in this population if retention, adherence and satisfaction rates each were ≥ 80%. We hypothesized that the intervention group would show a greater reduction in the body weight and caloric intake from baseline compared to the usual care group. If feasible and effective, the results of the study would serve as preliminary data to support the design of a larger, longer-term trial.

## 2. Materials and Methods

### 2.1. Study Design

This pilot study employed an assessor-blinded randomized controlled intervention design. All participants provided informed consent and those who completed the study were compensated with a $100 gift card. The West Virginia University Institutional Review Board approved the study procedures and the trial was registered at http://clinicaltrials.gov (NCT02938897).

### 2.2. Participants

Participants were patients of one of four collaborating PCPs across two office locations in Harrison County, WV who met inclusion and exclusion criteria. Participants were 40–70-year-old men who had a BMI ≥ 30 and a diagnosis of at least one of the following conditions: hypertension, hyperlipidemia, pre-diabetes and diabetes. Exclusion criteria included (1) having a diagnosis of kidney disease, liver disease, celiac disease or cancer (except skin or prostate), (2) having had major surgery or cardiovascular event (e.g., stroke or heart attack) in past six months, (3) using insulin, steroids, blood thinners requiring consistent vitamin K intake or anti-obesity medications, (4) drinking >2 alcoholic beverages daily, (5) already following a diet to lose weight or weight loss of ≥ 10 pounds in past 6 months and (6) did not have a home computer with high-speed internet service.

**Figure 1:**
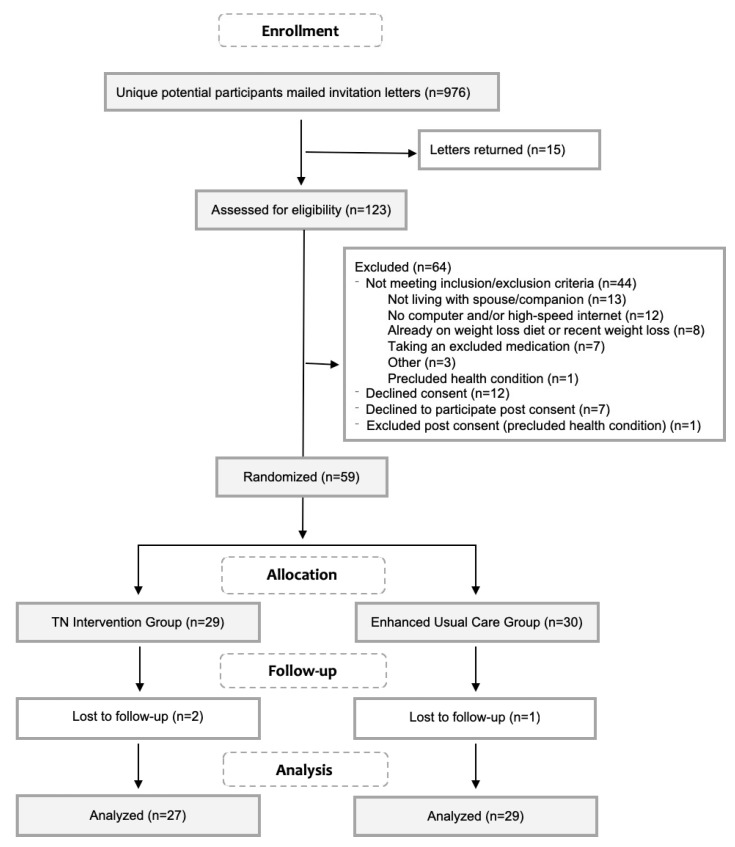
CONSORT flowchart of participant recruitment and study flow.

The CONSORT diagram in Figure 1 shows the flow of participants through the trial. Male patients 40–70 years old with a BMI ≥ 30 and a diagnosis of at least one of the qualifying diagnoses (*N* = 961) were mailed an invitation letter from their PCP. Of the 123 men screened for eligibility, 79 (64%) were eligible to participate and 67 of those eligible (85%) agreed to consent. Seven declined participation post consent and one was excluded due to an existing diagnosis that precluded participation. Recruitment took 8 weeks and ended when the desired number of participants electronically signed a combined HIPPA/consent form and scheduled their first in-person assessment visit. Fifty-nine men were randomly allocated to either the telenutrition intervention (*n* = 29) or the enhanced usual care (EUC) group (*n* = 30) by research personnel in the order they attended their first in-person session using a permuted block randomization in blocks of size two or four (block sizes chosen at random).

### 2.3. Telenutrition Intervention and Enhanced Usual Care (EUC) Groups

Participants were randomly assigned to either the telenutrition or the EUC group at the first in-person visit. EUC was usual care enhanced by the provision of educational materials to standardize care across physicians. Participants from both groups received an individualized caloric goal and diet-related educational materials and self-monitoring tools. Differences is treatment between groups pertained to the level of behavioral support provided. Those in the intervention group received weekly support from an RDN and a weight scale. Those in the EUC group did not receive behavioral support between in-person visits during the 12 weeks. However, the EUC participants were offered an in-person session with the RDN post study completion.

Participants from both groups met with researchers at a PCP office location at weeks 0, 6 and 12. At the baseline visit, weight and body composition were measured. All participants were prescribed a daily energy intake goal (500–750 kcal/day below their estimated requirement) by an RDN. Energy requirements were estimated using the Mifflin-St. Jeor equation with an energy factor based on self-reported activity levels. They were then provided a diet-related educational handbook which included topics such as building a healthy plate, making smart beverage choices and meal planning examples adapted from a diabetes plate method [15]. A brief (≤10 m) overview of the handbook contents was provided by the research RDN who performed anthropometric measures. Additionally, each participant was provided a binder containing self-monitoring tools [e.g. weekly weight log, fruit and vegetable tracker and a SMART (specific, measurable, achievable, relevant and time bound) goal planner]. All participants were asked to self-monitor their weight weekly using the provided paper log and to report the measures on the week 6 and week 12 online surveys. Additionally, they were asked not to change the duration or intensity of their baseline physical activity over the course of the study. General adherence was self-reported at subsequent in-person visits. At the final in-person session, participants were encouraged to increase physical activity post study completion for general and cardiovascular health and continued weight loss or weight loss maintenance.

Participants allocated to the intervention group received the same materials and brief nutrition education as described above in addition to weekly support from an RDN in the form of three virtual medical nutrition therapy (MNT) encounters at weeks one, five and nine and nine telephonic nutrition-coaching sessions at weeks 2–4, 6–8 and 10–12. During the encounters, the RDN was located at the university and participants were in their home or other self-selected location. During the videoconferencing sessions, the RDN provided individualized MNT including nutrition assessment, education and counseling based on the participants diet history, perceived barriers, medical diagnoses and laboratory values. The virtual encounters were delivered via a HIPAA compliant video-conferencing platform (Vidyo, Inc., Hackensack, NJ, USA) using a provided webcam (HD Pro Webcam C920, Logitech, Silicon Valley, CA, USA). The telephonic nutrition-coaching sessions were patient-led discussions on topics such as goal setting, self-monitoring weekly weight change and overcoming barriers to dietary adherence.

### 2.4. Measurements

#### 2.4.1. Demographic and Baseline Medical Data

Demographic data were collected via a self-administered online questionnaire distributed through RedCap (Research Electronic Data Capture) [16], a secure web-based application designed to support data capture for research studies hosted at the West Virginia Clinical and Translation Science Institute. Baseline medications, medical diagnoses and laboratory values were obtained from the medical record by the PCPs nursing staff were used to classify participants as having hypertension, pre-diabetes or diabetes and hyperlipidemia.

#### 2.4.2. Feasibility Measures

The feasibility assessment included measures of satisfaction and program adherence. Participants’ perception of program adherence and satisfaction each were assessed based on the response to a single question on the week 12 survey. To assess general program adherence and satisfaction, participants were asked to respond on a 5 point Likert scale to the question, how would you rate your overall adherence to the program? and to the statement, overall, I was satisfied with the program. The a priori criterion for feasibility for a larger trial included combining the two highest response categories (such as agree and strongly agree for satisfaction).

#### 2.4.3. Effectiveness Measures

Anthropometric and body composition data were collected at weeks 0, 6 and 12 at one of two PCP office locations. Participants were lightly clothed and without shoes and in a fasted but hydrated state (refraining from all food and beverages except water for 8–12 h prior). A single researcher blind to group allocation obtained all measurements using standard protocols. Measures were taken at the same time of day for each participant across visits to minimize within-subject variation; they were taken in duplicate and averages were used for analysis. Height was measured to the nearest 0.1 cm using the Seca 274 digital mobile stadiometer (Seca, Hamburg, Germany). Weight was measured to the nearest 0.1 kg with the scale automatically tared at 0.5 kg to adjust for clothing. Waist circumference (WC) was measured directly on the skin at the superior border of the iliac crest using a Gulick II tape measure. Weight and percent body fat were determined from segmental multi-frequency bioelectrical impendence analysis (MF-BIA) using the Seca medical Bioelectrical Composition Analyzer (mBCA) 514 (Seca, Hamburg, Germany) and Seca analytics 115 PC software (Seca, Hamburg, Germany). Body mass index (BMI) was calculated as weight (kg)/height (m^2^) and classified using World Health Organization classifications [17].

Diet was assessed using 4-day (3 weekdays and 1 weekend day) food records just prior to each visit at weeks 0, 6 and 12. Participants were provided written instruction and a copy of the Nutrition Data System for Research (NDSR) Foods Amounts Booklet [18] containing graphics to aide in portion size estimation. At the visit, study personnel reviewed the diet records with participants for completion and accuracy. Data from the food records were entered into NDSR software (version 2015, University of Minnesota Nutrition Coordinating Center) for analysis. Diet quality was assessed using the Healthy Eating Index-2015 index [19]. NDSR output data was transformed into the 12 HEI-2015 components according to the NDSR’s unpublished guide [20]. The Simple HEI Scoring Algorithm method for multiple days of intake data was applied [21]. The 12 component scores were summed for a total HEI score ranging from 0–100, with higher scores indicating better adherence to the 2015–2020 Dietary Guidelines for Americans.

### 2.5. Statistical Analysis

All statistical analyses were conducted using SAS software (version 9.4, SAS Institute Inc., Cary, NC, USA). Missing data was treated analytically with pairwise deletion. Baseline differences for groups were conducted on all demographics and select outcomes utilizing independent sample t-tests or exact Wilcoxon-Mann-Whitney U tests for continuous outcomes and chi-square or fisher’s exact tests for categorical variables, alpha = 0.05. Feasibility criteria of meeting a priori criterion of ≥ 80% self-rated adherence and satisfaction was examined by combining the two-positive agreement categories and tested with a one-sided test of the null proportion (0.80) along with confidence interval equivalence of the lower margin, alpha = 0.05. Follow-up analysis was conducted on the categories for relative weight change, examining group differences in proportions meeting 6-week and 12-week follow-up weight loss, using fisher’s exact testing to account for small cell sizes.

To account for the correlation amongst the repeated measurements on each participant, general linear mixed models (LMM) was the primary method of analysis for continuous outcomes. These models are designed to model correlations among observations on subjects (over time and/or within groups), are not required to meet many of the restrictive assumptions of a more traditional ANOVA approach (such as complete cases and compound symmetry) and are valid in the presence of missing at random (MAR) data [22] by use of the residual maximum likelihood (REML) approach to estimation and the Kenward-Roger approximation of the degrees of freedom. Various random effects and covariance estimates were compared using Akaike’s Information Criterion (AIC) to determine the best fitting model. The best fitting models for these models, presented, were an unstructured covariance matrix within the repeated statement. The hypothesis tests of interest involved the fixed effects portion of the model, namely the group by time interactions on the treatment outcomes. *P*-values are also presented for the group and time main effects. Significant *p*-values for all outcomes are presented for the fixed effects of group, time and group by time interaction effects.

The sample size calculation was based on the primary outcome of percent change in body weight at week 12, with a reported standard deviation between 2.5–4.0% Weight loss as low as 3% has been shown to be clinically meaningful [6]. To detect a 3% weight loss with 80% power and assuming the relative weight loss standard deviation is 3.5%, we estimated that 23 participants would be required to complete each arm of the study (46 in total).

## 3. Results

### 3.1. Baseline Characteristics

Baseline characteristics are shown in Table 1. Participants were, on average, 59.0 ± 7.7 years old. Overall, a majority were non-Hispanic white (96.6%) with more than a high school education (72.9%). Nearly half had household incomes >75,000 (49.1%) and most were employed full time (67.8%). All participants were obese and had a WC > 102 cm, an independent risk factor for CVD [23]; average BMI was 36.9 ± 5.9 kg/m^2^; the majority (64.4%) met criteria for stage 2 or 3 obesity. Nearly 90% had a diagnosis of dyslipidemia (hypertriglyceridemia or hypercholesterolemia), 83.1% had hypertension and 52.5% had either diabetes or pre-diabetes. There were no significant differences between treatment groups with respect to baseline characteristics. Most participants (90%) resided in counties classified as non-metro with urban populations of 20,000 or more (*n* = 40) or 2500 to 19,999 (*n* = 14) which are areas classified as ‘rural’ by the Federal Office of Rural Health Policy [24].

### 3.2. Feasibility: Retention, Satisfaction and Perceived Adherence

Three participants, two from the intervention group and one from the EUC group, withdrew from the study at or before week 6, yielding an overall retention rate of 94.9%. Overall, 88.5% (23/26; 95% exact CI: 0.70, 0.98) of participants in the intervention group rated their adherence to the program as good or very good. More than 92% (24/26; 95% exact CI: 0.75, 0.99) agreed or strongly agreed that they were ‘satisfied with the program.’ When tested against the a priori hypothesis that participants would meet or exceed 80% adherence and satisfaction for feasibility of the study, a one-sided test of difference from 0.80 was not significant (*p* = 0.14 for adherence and 0.06 for satisfaction) and when examining the difference in the lower margin of the confidence interval for equivalence testing we see significantly better than hypothesized proportion values (*p* = 0.016 for adherence and 0.001 for satisfaction). Overall compliance with the MNT sessions and health coaching calls was good. All participants in the intervention group who completed the study (*n* = 27) attended the three MNT sessions with the RDN. Of the nine health coaching calls made available, 18 participants completed 100% of calls; 3 completed 89% of calls, 3 completed 78% of calls and the remaining 3 completed 44–67% of calls.

### 3.3. Effectiveness: Change in Body Weight, Body Composition and Diet

Table 2 shows outcomes at week 0, 6 and 12. Notably, both groups had a substantial improvement over time for all measures (*p* < 0.0001). When examining the absolute and relative (percent of baseline) weight change over the 12-week period, participants in the intervention group lost a mean total of 8.3 kg (6.2% ± 3.5) and those in the EUC group lost a mean total of 5.0 kg (4.3% ± 4.3). At week 12, both groups had significant weight loss from baseline but there was insufficient evidence of a statistically significant difference between groups over time (absolute *p* = 0.111, relative *p* = 0.085), although all effects were in the pre-hypothesized direction of increased improvements for the intervention group relative to the EUC group. In addition, both groups had a substantial reduction over time in absolute energy intake (*p* < 0.0001) and improvements in overall diet quality (*p* < 0.0001). At weeks 6 and 12 respectively, the average reduction in caloric intake was 516.3 kcal (17.9% of baseline caloric intake ± 25.5) and 600.2 kcal (23.0% ± 20.2) in the intervention group and 473.0 kcal (21.3% ± 20.0) and 445 kcal (19.2% ± 21.1) in the EUC group. Group by time interaction effects were not significant but trended in the a priori direction (absolute *p* = 0.253, relative *p* = 0.198). Similar trends are noted for diet quality (time by group, *p* = 0.11).

Table 3 shows weight change relative to baseline weight over the 6-week and 12-week period. Notable group differences were seen at the 12-week time point for both increased proportions of losing at least 3% and 5% of their body weight among the intervention group. Seventy percent (19/27) of participants in the intervention group lost more than 5% of their body weight, versus 41.4% (12/29) in the EUC group (*p* = 0.035).

Table 4 shows the average HEI-2015 component scores stratified by group. Across both groups, significant time main effects were found for seven of the thirteen HEI components. For intervention and EUC groups, intakes improved for total fruit (*p* = 0.05), whole grains (*p* = 0.004) and fatty acid ratio (*p* = 0.002) and lowered for sodium (0.01), refined grains (*p* = 0.04), added sugars (*p* = 0.01) and saturated fats (*p* = 0.002). Significant group main effects were found for greens/beans, with the intervention group having lower scores than the EUC group at baseline (*p* = 0.013). Significant time by group interactions were found for greens/beans (*p* = 0.013), total fruit (*p* = 0.04) and whole fruit (*p* = 0.04) indicating that the time effects differed by groups, with greater improvements over time for the intervention group relative to the EUC group.

## 4. Discussion

This pilot study was designed to assess the feasibility and effectiveness of a telenutrition weight loss intervention compared to an EUC control group in 40- to 70-year-old men with multiple risk factors for CVD. Retention, adherence and satisfaction rates met our a-priori criterion for feasibility for a larger trial. We detected statistically significant weight loss from baseline to week 12 in both groups but a larger proportion of participants in the intervention group than the EUC group lost at least 5% of their baseline weight (70% vs. 41%, *p* = 0.035). Weight loss ≥5% is considered clinically significant weight reduction and if maintained, may decrease risk for development of, or improve the treatment of, obesity-related comorbidities regardless of the ending BMI [6]. Thus, based on the feasibility and effectiveness data, we determined that a larger longer-term trial would be feasible and warranted in this at-risk population.

Significant changes from baseline were detected in both groups for change in weight, waist circumference and percent body fat. Mean weight loss in this study was 8.3 kg or 7% of initial weight which approximates the average weight loss of 8 kg or 5–10% that was reported in a systematic review of result from 6-month intensive in-person lifestyle interventions (diet, physical activity and behavioral counseling) [6]. In other men-only weight loss trials, men in the intervention groups lost about 5% of baseline weight, 3.7 to 5.2 cm in waist circumference and 2.3% to 3.5% in % body fat over a 3-month period [25,26,27]. This is slightly less than the 7% weight loss and 6.8 cm mean reduction in waist circumference reported in the telenutrition intervention group in our study. Thus, tele-delivered programs prescribed for weight loss can yield similar results to in-person and technology-delivered interventions.

Diet quality was not reported in these other men-only studies [25,26,27] or in the systematic review. In this study, energy intake decreased while diet quality (HEI scores) improved significantly in both groups. Total diet quality scores at baseline were 51 out of 100 indicating diets that were generally ‘poor’ (HEI scores <51) to ‘needing improvement’ (HEI scores 51–80) [28]. Participants in the intervention and EUC groups significantly improved diet quality over the 12-week trial by 40% and 25%, respectively. The changes included significant reductions in intakes of saturated fat, added sugars and sodium. The intervention group had a significantly larger improvement in intakes at week 12 than the EUC group in three of component categories: whole fruit, total fruit and beans and greens. Improvements in diet quality are clinically important; higher quality diets have been inversely associated with overall mortality [29], and improvements in diet quality scores over time have been associated with lower CVD risk [30].

Despite having statistically significant improvements in all primary and secondary outcome measures, the changes were not better in the intervention group than the EUC in most outcomes as we had hypothesized. We likely did not detect a between group difference for a few reasons. First, the EUC was not a ‘no treatment’ control; the enhancement of usual care included a prescription of a moderate energy reduction in diet and educational materials. Additionally, all participants were asked to self-monitor their weekly weight and to return for in-person weigh-ins every 6 weeks. Both the self-monitoring and accountability may have contributed to weight reduction in both groups. A recent systematic review of behavioral weight loss programs reported a mean difference in weight loss between intervention and control groups to be 2.9 kg which was close to the 3.3 kg between group difference in this study [31]. Second, the change in diet and weight among men in the EUC group may reflect motivation of those who chose to enroll in the weight loss trial upon invitation. This is true of most behavior-based trials; when there is a degree of self-selection, participants are likely to be more motivated to change their behaviors than individuals not enrolling in the trials [31]. However, this study demonstrated that middle-aged and older men can lose weight with varying degrees of support, but that a larger proportion lose an amount that is considered clinically significant with weekly behavioral support from an RDN at week 12. While high intensity programs (weekly for the first 3 to 6 months) are recommended as gold-standard treatment [6,32], similar results, at least in the short-term, are feasible with less intense support. Adequately powered comparative effectiveness trials are needed to determine the most cost-effective level of intensity.

This study contributes to the limited research on weight loss in men and primary-care-referred telenutrition weight-loss programs. Among the strengths of this study are the use of a randomized study design, high retention rate, outcome assessor blind to group allocation and the inclusion of diet quality outcomes. There are also notable limitations to this study. First, the study was designed to determine feasibility and short-term effectiveness to inform the design of a larger trial. A longer-term trial is needed to determine if the dietary changes and weight loss are sustainable over time. Second, the study consisted mainly of non-Hispanic whites. While the racial and ethnic homogeneity is representative of the population in the state (94%), generalizability to racially and ethnically diverse groups is unknown. Third, our sample is of higher income and educational level than the state average. The inclusion criteria requiring access to a computer and high-speed internet may have limited low-income men or those with low computer literacy from participating. However, in non-Hispanic white men, there is no difference in obesity rates by income levels; most obese men are not low income [33]. Thus, middle-aged men in the state are a health disparate subpopulation regardless of income and education level. Nevertheless, the sample may not represent men across sociodemographic backgrounds and efforts should be made in future studies to include men with lower incomes and educational levels. It should also be noted, that men seeking primary care likely differ from those who do not seek medical care; thus, the results cannot be generalized to include those who do not seek primary care. Fourth, this was a diet only trial. Longer-term studies should include a physical activity component. Last, we did not measure intermediate health outcomes (i.e., lipids and blood pressure) related to the dietary changes and weight loss or conduct a cost analysis. Future longer-term studies should include measurements of intermediate health outcomes, a post-intervention follow-up, and a cost-benefit analysis.

## 5. Conclusions

This study has demonstrated that telenutrition programs are effective in the short term in reducing caloric intake and body weight and improving diet quality in middle-aged and older men at risk for CVD. Based on good retention, satisfaction and adherence rates a larger trial in this at-risk population is feasible. Thus, the results of the study will serve as preliminary data to support the design of a larger longer-term trial to determine if the results are sustainable over time.

## Figures and Tables

**Table 1:** Baseline characteristics.

Characteristic	Telenutrition Intervention (*n* = 29)	Enhanced Usual Care (*n* = 30)	*p*-Value
Age, years	58.6 ± 8.1	59.3 ± 7.4	0.71
Weight, kg	114.9 ± 20.6	115.6 ± 21.8	0.90
Height, cm	176.4 ± 5.3	176.9 ± 5.8	0.75
Body Mass Index, kg/m^2^	36.9 ± 6.4	36.8 ± 5.6	0.96
Race: White	29 (100.0)	28 (93.3)	1.00
Ethnicity: Non-Hispanic	29 (100.0)	28 (93.3)	1.00
Household Income			0.80
<35 k	3 (10.3)	3 (10.0)	
35k–50 k	2 (6.9)	3 (10.0)	
50k–75 k	6 (20.7)	6 (20.0)	
>75 k	16 (55.2)	13 (43.3)	
Did not respond	2 (6.9)	5 (16.7)	
Education			0.84
High school graduate	9 (31.0)	7 (23.3)	
Some college	7 (24.1)	7 (23.3)	
College graduate	13 (44.8)	16 (53.3)	
Obesity Classification (BMI)			0.92
Class 1 (BMI 30.0–34.9)	11 (37.9)	10 (33.3)	
Class 2 (BMI 35.0–39.9)	13 (44.8)	15 (50.0)	
Class 3 (BMI 40.0+)	5 (17.3)	5 (16.7)	
Comorbidities			
Pre-diabetes/diabetes	12 (41.4)	19 (63.3)	0.12
Hyperlipidemia	26 (89.7)	27 (90.0)	1.00
Hypertension	25 (86.2)	24 (80.0)	0.73
Number of comorbidities			0.70
1	6 (20.7)	4 (13.3)	
2	12 (41.4)	12 (40.0)	
3	11 (37.9)	14 (46.7)	

Values reported as mean ± SD or *n* (%). *p*-values were calculated with *t*-tests or exact Wilcoxon-Mann-Whitney U for continuous variables and chi-square or fisher’s exact for categorical.

**Table 2:** Primary and secondary outcomes variables at baseline, week 6 and week 12 by treatment group.

Outcome	Week	Telenutrition Intervention	Enhanced Usual Care	*p*-Value		
				Group	Time	Group × Time
Weight, kg	Baseline	114.9 ± 20.6	115.6 ± 21.8	0.997	<0.0001	0.111
	Week 6	109.3 ± 18.8	112.4 ± 20.8			
	Week 12	106.6 ± 19.0	110.6 ± 20.4			
Waist Circumference, cm	Baseline	123.5 ± 12.9	125.8 ± 13.2	0.605	<0.0001	0.128
	Week 6	119.0 ± 12.0	123.3 ± 13.4			
	Week 12	116.7 ± 12.4	121.3 ± 13.9			
Body Fat, %	Baseline	38.2 ± 4.9	39.6 ± 4.7	0.297	<0.0001	0.772
	Week 6	36.6 ± 5.0	38.5 ± 5.4			
	Week 12	35.7 ± 5.3	37.7 ± 6.1			
Energy intake, kcal	Baseline	2194.1 ± 578.2	2138.3 ± 623.7	0.399	0.0001	0.253
	Week 6	1677.8 ± 462.9	1665.3 ± 553.2			
	Week 12	1593.9 ± 424.3	1693.3 ± 480.8			
Diet quality, 0–100	Baseline	51.0 ± 10.9	51.1 ± 14.0	0.629	<0.0001	0.110
	Week 6	70.6 ± 14.3	61.2 ± 15.6			
	Week 12	71.3 ± 13.9	63.9 ± 14.8			

Values are means ± standard deviations.

**Table 3:** Weight change relative to baseline weight at weeks 6 and 12.

Outcome	Telenutrition Intervention	Enhanced Usual Care	*p* Value ^a^
**Week 6**	**(*n* = 27)**	**(*n* = 30)**	
Gained	1 (3.7)	2 (6.7)	0.40
Lost 0–2.9%	12 (44.4)	17 (56.7)	
Lost 3–4.9%	5 (18.5)	6 (20.0)	
Lost 5–9.9%	9 (33.3)	4 (13.3)	
Lost ≥ 10%	0 (0.0)	1 (3.33)	
Lost ≥ 5%	9 (33.3)	5 (16.7)	0.22
**Week 12**	**(*n* = 27)**	**(*n* = 29)**	
Gained	2 (7.4)	4 (13.8)	0.25
Lost 0–2.9%	3 (11.1)	9 (31.0)	
Lost 3–4.9%^b^	3 (11.1)	4 (13.8)	
Lost 5–9.9%	15 (55.6)	9 (31.0)	
Lost ≥ 10%	4 (14.8)	3 (10.3)	
Lost ≥ 5%	19 (70.4)	12 (41.4)	0.035

Data presented as *n* (%). ^a^ *p* value presented for fisher’s exact.

**Table 4:** Diet quality Healthy Eating Index 2015 component scores at weeks 0, 6 and 12 by group.

Outcome	Max Score	Week	Telenutrition Intervention	Enhanced Usual Care	*p*-Value		
					Group	Time	Group × Time
Total Vegetables	5	Baseline	3.62 ± 1.3	3.70 ± 1.4	0.471	0.203	0.206
		Week 6	4.56 ± 0.9	4.38 ± 1.1			
		Week 12	4.69 ± 0.8	4.23 ± 1.1			
Greens and Beans	5	Baseline	1.15 ± 1.6	2.51 ± 2.2	0.013	0.411	0.013
		Week 6	3.56 ± 2.0	2.83 ± 2.0			
		Week 12	3.55 ± 1.9	2.95 ± 2.3			
Total Fruit	5	Baseline	1.77 ± 1.4	1.69 ± 1.5	0.333	0.050	0.040
		Week 6	2.92 ± 1.5	2.40 ± 1.7			
		Week 12	3.71 ± 1.7	2.51 ± 1.9			
Whole Fruit	5	Baseline	2.43 ± 1.9	2.60 ± 2.0	0.297	0.070	0.037
		Week 6	3.72 ± 1.6	3.29 ± 1.9			
		Week 12	4.22 ± 1.4	3.31 ± 1.9			
Whole Grains	10	Baseline	3.97 ± 3.1	4.06 ± 3.6	0.930	0.004	0.580
		Week 6	7.08 ± 3.3	5.44 ± 3.5			
		Week 12	6.42 ± 3.7	5.49 ± 3.7			
Dairy	10	Baseline	5.38 ± 2.5	5.06 ± 2.9	0.877	0.592	0.807
		Week 6	4.95 ± 3.0	5.46 ± 2.4			
		Week 12	5.33 ± 2.8	4.82 ± 3.0			
Total Protein Foods	5	Baseline	4.82 ± 0.6	4.80 ± 0.7	0.835	0.124	0.993
		Week 6	5.00 ± 0.0	4.86 ± 0.6			
		Week 12	4.93 ± 0.3	4.99 ± 0.1			
Seafood and Plant Protein	5	Baseline	2.97 ± 2.1	3.60 ± 1.9	0.269	0.604	0.092
		Week 6	4.65 ± 1.1	3.51 ± 2.0			
		Week 12	3.89 ± 1.9	3.32 ± 2.2			
Fatty Acid Ratio	10	Baseline	4.28 ± 2.8	3.41 ± 2.2	0.116	0.002	0.228
		Week 6	6.45 ± 3.2	5.04 ± 2.9			
		Week 12	5.55 ± 3.4	5.81 ± 2.8			
Sodium	10	Baseline	2.57 ± 2.8	2.30 ± 2.6	0.802	0.013	0.485
		Week 6	3.30 ± 3.0	3.31 ± 3.2			
		Week 12	4.95 ± 3.2	4.16 ± 3.5			
Refined Grains	10	Baseline	6.45 ± 3.3	5.94 ± 3.6	0.474	0.039	0.643
		Week 6	8.51 ± 2.3	6.65 ± 3.4			
		Week 12	8.53 ± 2.3	7.33 ± 3.2			
Added Sugar	10	Baseline	7.60 ± 2.5	7.76 ± 2.9	0.894	0.012	0.720
		Week 6	9.29 ± 1.2	8.74 ± 2.2			
		Week 12	9.41 ± 1.0	8.99 ± 2.1			
Saturated Fats	10	Baseline	4.03 ± 2.6	3.65 ± 2.4	0.298	0.002	0.493
		Week 6	6.66 ± 2.7	5.26 ± 3.4			
		Week 12	6.12 ± 2.8	6.02 ± 3.2			

Diet quality was measured using the Healthy Eating Index 2015. Data presented as means and standard deviations. Higher scores indicate better adherence to the 2015 Dietary Guidelines for the food component (i.e., lower intakes of sodium, refined grains, added sugars and saturated fats and higher intakes of all other components). Significance testing conducted via linear mixed modeling.
